# Cumulative Radiation Exposure Post Aneurysmal Subarachnoid Haemorrhage

**DOI:** 10.1007/s00062-025-01513-8

**Published:** 2025-03-31

**Authors:** Shreepad H. Asundi, Mark P. Plummer, Krishnaswamy Sundararajan, Gerry O’Callaghan, Palash Kar, Alistair Jukes, Chris M. Boyd, Weitong Chen, Chang Dong, Timothy Webber

**Affiliations:** 1 Department of Intensive Care, Royal Adelaide Hospital, 5000 Adelaide, South Australia Australia; 2 Adelaide Medical School, The University of Adelaide. Adelaide, South Australia 5000, Adelaide, Australia; 3 Discipline of Acute Care Medicine, University of Adelaide, Adelaide, Australia; 4 Faculty of Health Sciences, School of Public Health, The University of Adelaide, South Australia 5000, Adelaide, Australia; 5 Department of Neurosurgery, Royal Adelaide Hospital, South Australia 5000, Adelaide, Australia; 6 Medical Physics & Radiation Safety, South Australia Medical Imaging, South Australia 5000, Adelaide, Australia; 7 Allied Health &Human Performance, University of South Australia, South Australia 5000, Adelaide, Australia; 8 Australian Institute for Machine Learning, The University of Adelaide, 5005 Adelaide, South Australia Australia; 9 School of computer and mathematical Sciences, The University of Adelaide, South Australia 5005, Adelaide, Australia; 10 School of Biomedicine, The University of Adelaide, 5000 Adelaide, South Australia Australia

**Keywords:** Subarachnoid haemorrhage, Ionising radiation, Lens, Neurosurgery

## Abstract

**Background:**

Patients with aneurysmal subarachnoid haemorrhage (aSAH) often receive multiple radiation based diagnostic studies. Cumulative radiation exposure has been associated with long term health consequences from both dose dependent deterministic harm and increased risk of developing adverse events in a non-dose dependant manner i.e. stochastic harm.

**Objective:**

The objective was to calculate cumulative radiation exposure in the acute phase after aSAH.

**Design, Setting, Participants, Main Outcome Measures:**

Single centre retrospective, observational study of consecutive adult patients admitted to the ICU for management of aSAH over five years. Organ and effective radiation doses were determined using institution specific conversion coefficients based on scanner radiation output metrics for all computed tomography imaging and fluoroscopy examinations. Calculated patient doses for the duration of the hospital admission were determined using National Cancer Institute radiation dosimetry tools.

**Results:**

A total of 276 patients met the inclusion criteria; 180 females (65%), mean (SD) age 56 (13) years. There were 222 (80%) patients who survived to hospital discharge. The median [IQR] effective cumulative radiation dose was 17.7 [9.7–30.5] mSv. Twenty-one patients (8%) received an effective dose > 50 mSV consistent with potentially harmful ionising radiation exposure. In 162 patients (59%), the equivalent radiation dose to the lens of the eye exceeded the 500 mSv threshold for radiation induced damage.

**Conclusion:**

Survivors of aSAH are exposed to high levels of medical radiation. The eyes are particularly at risk with most patients exposed to levels known to induce lens damage. This highlights the importance of strategies to reduce incidental and cumulative medical radiation exposure in this population.

**Supplementary Information:**

The online version of this article (10.1007/s00062-025-01513-8) contains supplementary material, which is available to authorized users.

## Introduction

Diagnosis and management of aSAH depends heavily on radiation that has enough energy to break an electron away from an atom, known as ionising radiation. Patients are repeatedly exposed to ionising radiation from serial studies including non-contrast computed tomography of the brain (CTB), CT angiography of cerebral arteries (CTA), CT perfusion (CTP), digital subtraction angiography (DSA) and plain x‑rays of the chest.

Above certain organ-specific thresholds, harm from ionising radiation occurs from direct tissue damage in a dose dependent manner; this is known as deterministic harm and includes skin damage, cataracts, and sterility [1–3]. Harm from ionising radiation can also be from stochastic effects whereby the dose of radiation increases the probability of developing adverse events but not the severity of the event itself; stochastic effects include solid organ tumours and haematological malignancy [4]. The International Commission on Radiological Protection (ICRP) reports an age and sex averaged cancer risk of 5.5% per Sievert (Sv) for the general population [5]. Extrapolated linearly to the age of 85, a whole-body CT scan increases the lifetime risk of cancer from 40% to 40.05% [6].

Patients with aSAH receive greater ionising radiation exposure compared to many other critically ill patient cohorts due to the protracted nature of their critical illness and the reliance on radiation dependent imaging modalities for the diagnosis and management of the acute condition and its complications. Observational studies from the United States have demonstrated that up to one-third of patients admitted to ICU with aSAH are exposed to potentially harmful radiation, arbitrarily defined as > 50 mSv [7, 8]. There is marked heterogeneity in the management of patients with aSAH globally [9], and radiation exposure has never been quantified in aSAH patients managed in Australian Critical Care Units. The aim of this study was to quantify the cumulative radiation effective dose for patients admitted to an Australian ICU with aSAH. Given the susceptibility of the eye to deterministic harm, the secondary aim was to quantify the cumulative radiation equivalent dose to the lens.

## Methods

We conducted a single-centre, retrospective, observational study of consecutive patients admitted to the Royal Adelaide Hospital Intensive Care Unit with a diagnosis of aSAH. The Royal Adelaide Hospital is a large mixed medical-surgical intensive care unit providing quaternary referral neurosurgical critical care. The study was conducted according to the Strengthening and Reporting of Observational Studies in Epidemiology (STROBE) statement [10]. The project was approved by the Central Adelaide Local Health Network Human Research Ethics Committee (CAHLN HREC) with reference number 17,482. A waiver of consent was granted by the HREC due to the retrospective nature of the study.

We screened all adult patients over a five-year period (Oct 2017 to Sep 2022) admitted to the ICU with a principal diagnosis of subarachnoid haemorrhage recorded on the ANZICS (Australian and New Zealand Intensive Care Society) Adult Patient Database [11]. All patients regardless of outcome were included in the study. Patients with non-aneurysmal subarachnoid haemorrhage attributed to infection, trauma or arteriovenous malformations were excluded.

Demographic, processes of care, and length of stay data were recorded from the ANZICS Adult Patient Database. Patient medical records were then reviewed to extract the following variables: the World Federation of Neurosurgical Surgeons (WFNS) grade [12], location of aneurysm, treatment modality (conservative, endovascular coiling, neurosurgical clipping, stenting), and complications including rebleeding, acute hydrocephalus necessitating insertion of an external ventricular drain, cerebral vasospasm and ventriculitis. Posterior circulation aneurysms refers to those arising from the vertebral or basilar arteries, while the remainder were considered to be anterior circulation aneurysms.

Cerebral vasospasm was diagnosed radiologically from CTA and DSA reports. Admission CT scans were evaluated and graded according to the modified Fisher scale [13]. In addition, we recorded the following imaging and procedures that exposed patients during the acute inpatient stay to medical radiation: CT, CTA, CTP, and DSA. Planar radiography (‘x-ray’) produces a relatively low dose of radiation (< 0.1 mSv) [14] that were considered trivial within calculation uncertainties and were excluded from dose calculations.

An automated system was used for collecting dosage parameters for all CT episodes (OpenREM Version 0.10.0 (Cardiff, United Kingdom)). Automated scan parameter collection was not available for DSA imaging, for which radiation dose summary data were manually extracted including total dose and individual parameters for exposure runs. As the tube geometry and machine parameters were unavailable for individual screening events, the dose distribution of screening was taken to match that of the exposure runs and used to calculate organ and effective coefficients. CT scans were performed using either the Toshiba Aquilion One Vision with V8.3 software or the Siemens SOMATOM Force. Angiographies were performed with the Philips Allura FD 20 10 BiPlane with Interventional Workspot software Version R1.5.1.1047.

For each of the four most frequently used CT protocols (head CT, head CTA, head CTP stroke diagnosis and head CTP stroke follow-up) a radiology medical physicist calculated the organ and effective doses for twenty randomly sampled patients, using the NCIDose software suite (National Cancer Institute, United States government). Based on the average normalised dose for these patients a local equivalent to the International Commission on Radiological Protection (IRCP) ‘k-factor’ was developed for each CT examination (mSv/[mGy.cm]), and DSA procedure (mSv/[Gy.cm^2^]). Cumulative radiation doses were calculated for all studies undertaken during the incident hospital admission.

## Statistics

Descriptive analyses were performed using SPSS Version 22 on all baseline variables and are reported as mean and standard deviation, median and interquartile range or frequency and percentages, as appropriate. Normality was assessed per Kolmogorov Smirnov test [15]. Differences between groups were compared with Kruskwal-Wallis test [16]. A *P* value of 0.05 was considered statistically significant.

## Results

There were 498 patients admitted to the Intensive Care Unit with a diagnosis of SAH over the study period. After excluding non-aneurysmal, traumatic, and arterio-venous malformations causes for subarachnoid blood, 276 patients with aSAH were included in the analysis. In total, 222 (80%) patients survived hospital discharge. Demographic, clinical and outcome data are presented in Table 1. Aneurysm location and management are presented in Table 2 and 3, respectively. Frequency of radiation exposure episodes per patient are presented in Table 4.

**Table 1:** Patient characteristics

Number of patients	276
Age (years)	56 (13)
Female	180 (65%)
ICU length of stay (hours)	113 [46–227]
Hospital length of stay (hours)	377 [303–575]
ANZROD	0.14 ± 0.17
WFNS Grade	
I–III	74%
IV‑V	26%
mFisher grade	
1–2	26%
3–4	74%
Hospital Mortality	54 (20%)

Data are number (percentage), mean (standard deviation) and median [interquartile range] *ANZROD* Australian and New Zealand Risk of Death admission model, *ICU* intensive care unit, *WFNS* World Federation of Neurological Surgeons.

**Table 2:** Aneurysm location

Aneurysm location	Number (%)
ACOM	86 (31%)
MCA	49 (18%)
Multiple	43 (16%)
PCOM	40 (14%)
ICA	28 (10%)
Basilar	22 (8%)
Vertebral	8 (3%)

*ACOM* Anterior communicating artery, *MCA* Middle cerebral artery, *PCOM* Posterior communicating artery, *ICA* Internal carotid artery

**Table 3:** Aneurysm Treatment

Management	*N* (%)
Coiling	158 (57)
Clipping	77 (28)
Conservative	25 (9)
Clip and coil	14 (5)
Flow diverting stent	2 (< 1)

**Table 4:** Radiological studies performed per patient

Study	Median [IQR] examinations
CTP	2 [1–5]
DSA	2 [1–2]
CTB	1 [0–2]
CT angiography	1 [1–1]
CTPA	0 [0–0]
CT Chest	0 [0–0]
CT abdomen	0 [0–0]

*CT* computed tomography, *CTP* CT perfusion, *CTB* CT brain, *CTPA* CT pulmonary angiogram, *DSA* Digital subtraction angiography

### Total Effective Radiation Dose

The median [IQR] total effective radiation dose was 17.7 [9.7–30.5] mSv; this included 21 patients (8%) with exposure greater than the 50 mSv threshold for ‘potentially harmful ionising radiation’, and one patient with exposure > 100 mSv (Table 5). There was no difference in total effective radiation dose between aneurysms secured by clipping versus coiling; 18.5 [10.3–33.6] vs. 20.1 [11.5–32.2] mSv; *P* = 0.67. There was no difference in total effective radiation dose between aneurysms located in the anterior versus posterior circulations; 18.0 [10.0–34.1] vs 15.5 [9.1–25.8] mSv; *P* = 0.15. There was no difference in radiation exposure between those that survived vs those that died; 19.16 [3.20–35.99] vs 18.05 [11.24–29.86] mSv; *P* = 0.88. Investigations per participant are presented in Supplementary Fig. 1.

**Table 5:** Total Effective radiation dose

Total effective radiation dose (mSv)	Number (%)
< 25	187 (68)
25–50	68 (25)
50–100	20 (7)
> 100	1 (0.3)

### Lens Equivalent Radiation Dose

The median [IQR] equivalent radiation dose delivered to the lens of the eye post aSAH was 629.2 [312–1226.1] mSV. This included 162 patients (59%) with an equivalent dose that exceeded the 500 mSv deterministic threshold for radiation induced lens damage, with one patient in whom the dose exceeded 4000 mSv. There was no difference in lens equivalent radiation between aneurysms secured by clipping versus coiling; 866.7 [367.6–1532.2] vs 691.2 [386.2–1246.9] mSv; *P* = 0.29. There was no difference in lens equivalent radiation dose between aneurysms located in the anterior or posterior circulations, 663.5 [297.4–1289.8] vs 530.9 [285.2–1017.3] *p* < 0.05. See Supplementary Table 1 for radiation dose per scan and equivalent radiation doses to other organs are presented in Supplementary Fig. 2.

## Discussion

In this retrospective observational study of patients admitted to an Australian intensive care unit with aSAH, we report lower levels of medical radiation exposure compared to international data. However, most patients receive incidental exposure to the lens of the eye above the critical threshold for deterministic radiation injury that induces cataracts. In total, 8% of patients were exposed to a total effective radiation dose that exceeded the recognised threshold for Potentially Harmful Radiation Exposure (PHIRE) (> 50 mSV). This is the first study to quantify radiation exposure post aSAH in the Australian setting. While there is no minimum safe threshold for stochastic effects, such as cancer, the median [IQR] cumulative radiation exposure of 17.7 [9.7–30.5] mSv was lower than reported internationally. Two US based single-centre retrospective observational studies of patients with aSAH report mean (SD) cumulative radiation doses of 45.8 (27.1) mSV (*n* = 192), and 37.1 (23.2) mSV (*n* = 146) respectively [7, 17]. Similar levels of cumulative radiation exposure were reported in a German observational study of 268 patients with aSAH; mean (SD) 39.9 ± 30.4 mSv [18].

Radiation dose depends on several factors, including scanner geometry, collimator design, slice spacing and image quality requirements. CT scans in our institution use dose modulation, iterative or artificial intelligence-reconstruction, and size-based dosing to reduce radiation dose while maintaining diagnostic quality. These factors may be responsible for the difference in radiation exposure in our study population compared to other studies.

The lens of the eye is exquisitely sensitive to radiation induced injury [19, 20], with the primary concern being cataracts that develop in a dose dependent fashion above a radiation threshold of 500 mSv [3, 21, 22]. Two nationwide population-based studies in Taiwan have demonstrated that repeat exposure of the head and neck to ionising radiation approximately doubles the lifetime risk of cataracts [23, 24]. Neither of these studies calculated the radiation dose to the lens of eye. For the first time, we report a very high equivalent radiation dose to the lens of eye in critically ill patients managed in ICU with aSAH. This is of particular importance given the relatively young age of this cohort and high proportion of survivors (86%). These data highlight the importance of strategies to minimise incidental irradiation of the eye in the routine management of patients with aSAH. There are two well described strategies to reduce lens exposure; gantry tilt and bismuth shielding, of which gantry tilt has been shown to be more effective [25]. When gantry tilt angle is aligned with the supraorbital line, the lens is not exposed to the primary beam and the radiation dose is reduced by more than 75% [26]. The global reduction of tube current, the use of iterative reconstruction and the application of organ-based tube current modulation have also shown benefit in reducing the radiation dose to the lens of the eye [27, 28]. These proposed techniques are all easily implemented but not widely adopted. Finally, while not routinely used at our institution, non-radiation-based modalities like trans-cranial doppler and magnetic resonance angiography (MRA) could be used to reduce radiation exposure. There are however disadvantages to these modalities. Transcranial doppler is less sensitive and specific at diagnosing symptomatic vasospasm than CTA, and does not provide information on cerebral perfusion but can be done at the bedside sequentially throughout a patient’s stay [29]. MRA requires longer imaging times than CT imaging and over estimates stenotic lesions [30, 31].

There are several limitations in addition to the retrospective observational design. Firstly, the radiation doses were not measured directly using a dosimeter and we cannot exclude that the calculations used over or under-estimated the true radiation dose. Secondly, the study was undertaken in a single-centre that does not have a neurosurgical high-dependency unit or step-down unit and we suspect that we admit lower acuity haemorrhages than other institutions; accordingly, the external validity may be limited even within Australia due to heterogeneity of clinical management [32]. Moreover, our institution does not have a standard operating procedure for the diagnosis and management of aSAH making direct comparisons between institutions difficult. In addition, there was no formal screening for radiation sequelae such as hair loss during the hospital admission and these data were not collected.

While we report levels associated with stochastic harm and deterministic harm to the lens of the eye, we are unable to demonstrate causation which would require a long-term prospective study. Finally, we only calculated the radiation dose during the index acute hospital admission. This patient cohort will be exposed to subsequent medical ionising radiation as a part of follow-up in the years post the sentinel bleed. Accordingly, these values under-represent the lifetime radiation exposure following aneurysm rupture.

## Conclusions

Patients with aSAH managed in an Australian ICU are exposed to lower levels of ionising radiation than reported internationally. This is likely multifactorial including, lower patient acuity along with dose modulation, iterative and artificial intelligence reconstruction, and size-based dosing. However, incidental exposure to the eyes is concerning with most patients exposed to levels known to induce cataracts. Implementation of simple strategies to reduce lens exposure in this population is a priority.

## Supplementary Information

Supplementary Fig. 1: Boxplot of number of investigations by category and ICU outcome. CT = Computed Tomography, CTPA = Computed Tomography Pulmonary Angiogram; Count = Number of investigations performed per patient

Supplementary Fig. 2: Boxplot of highest radiation exposure by body part. Median > 400 = body parts with median radiation exposure > 400 millisieverts; Y axis = median radiation exposure, x axis = body part exposed

Supplementary Table 1: Number of different types of investigations performed and the DLP for each scan type. COW = Circle of Willis, DLP = Dose length Product

## Data Availability

The data that support the findings of this study are available on scientifically justified request from the corresponding author.
