# Supplementary material for: Cumulative Radiation Exposure Post Aneurysmal Subarachnoid Haemorrhage
Source: Clin Neuroradiol. 2025 Mar 31;35(3):559–64. doi: 10.1007/s00062-025-01513-8 (PMC12454507; doi:10.1007/s00062-025-01513-8)
Supplement: Supplementary file 1 — Supplementary Fig. 1: Boxplot of number of investigations by category and ICU outcome. CT = Computed Tomography, CTPA = Computed Tomography Pulmonary Angiogram; Count = Number of investigations performed per patient [file 62_2025_1513_MOESM1_ESM.docx]

Supplementary Figure 1: Boxplot of number of investigations by category and ICU outcome


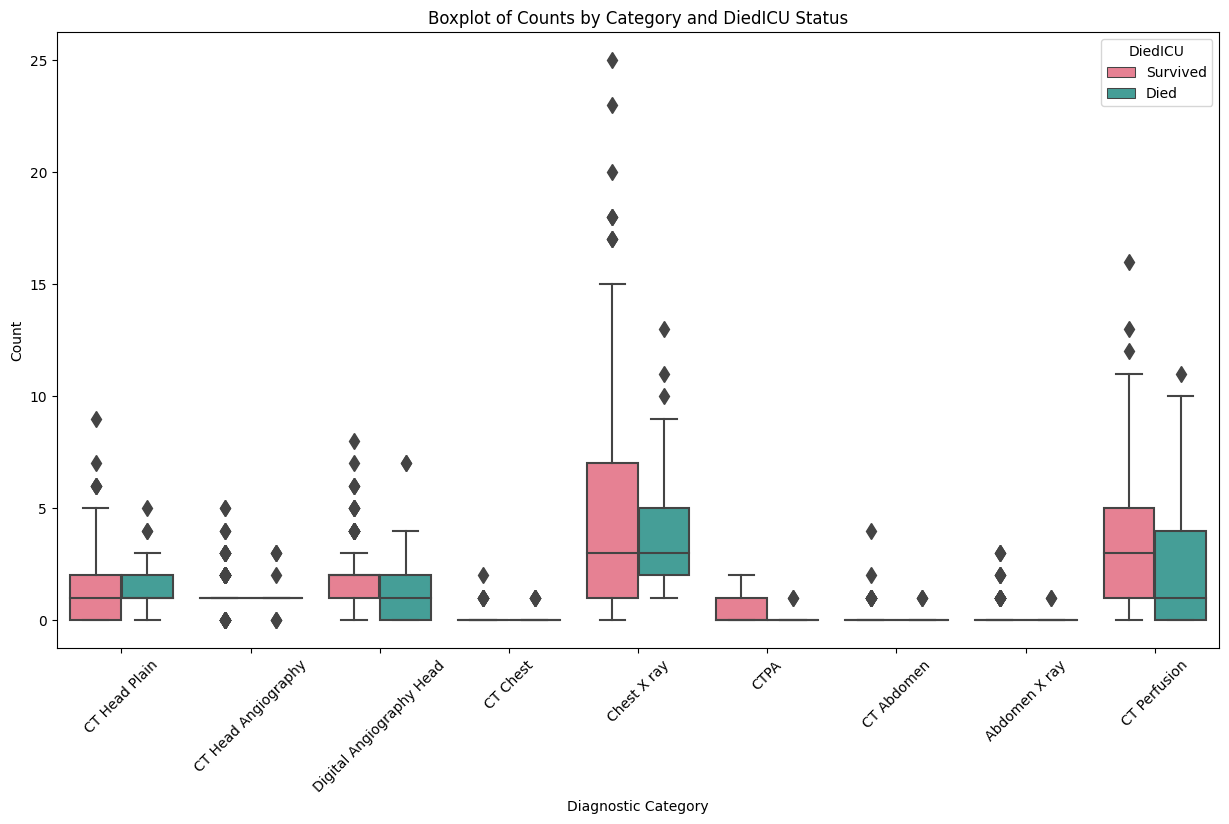


CT = Computed Tomography, CTPA = Computed Tomography Pulmonary Angiogram

Count = Number of investigations performed per patient
