# Supplementary material for: Cumulative Radiation Exposure Post Aneurysmal Subarachnoid Haemorrhage
Source: Clin Neuroradiol. 2025 Mar 31;35(3):559–64. doi: 10.1007/s00062-025-01513-8 (PMC12454507; doi:10.1007/s00062-025-01513-8)
Supplement: Supplementary file 2 — Supplementary Fig. 2: Boxplot of highest radiation exposure by body part. Median > 400 = body parts with median radiation exposure > 400 millisieverts; Y axis = median radiation exposure, x axis = body part exposed [file 62_2025_1513_MOESM2_ESM.docx]

Supplementary Figure 2: Boxplot of highest radiation exposure by body part


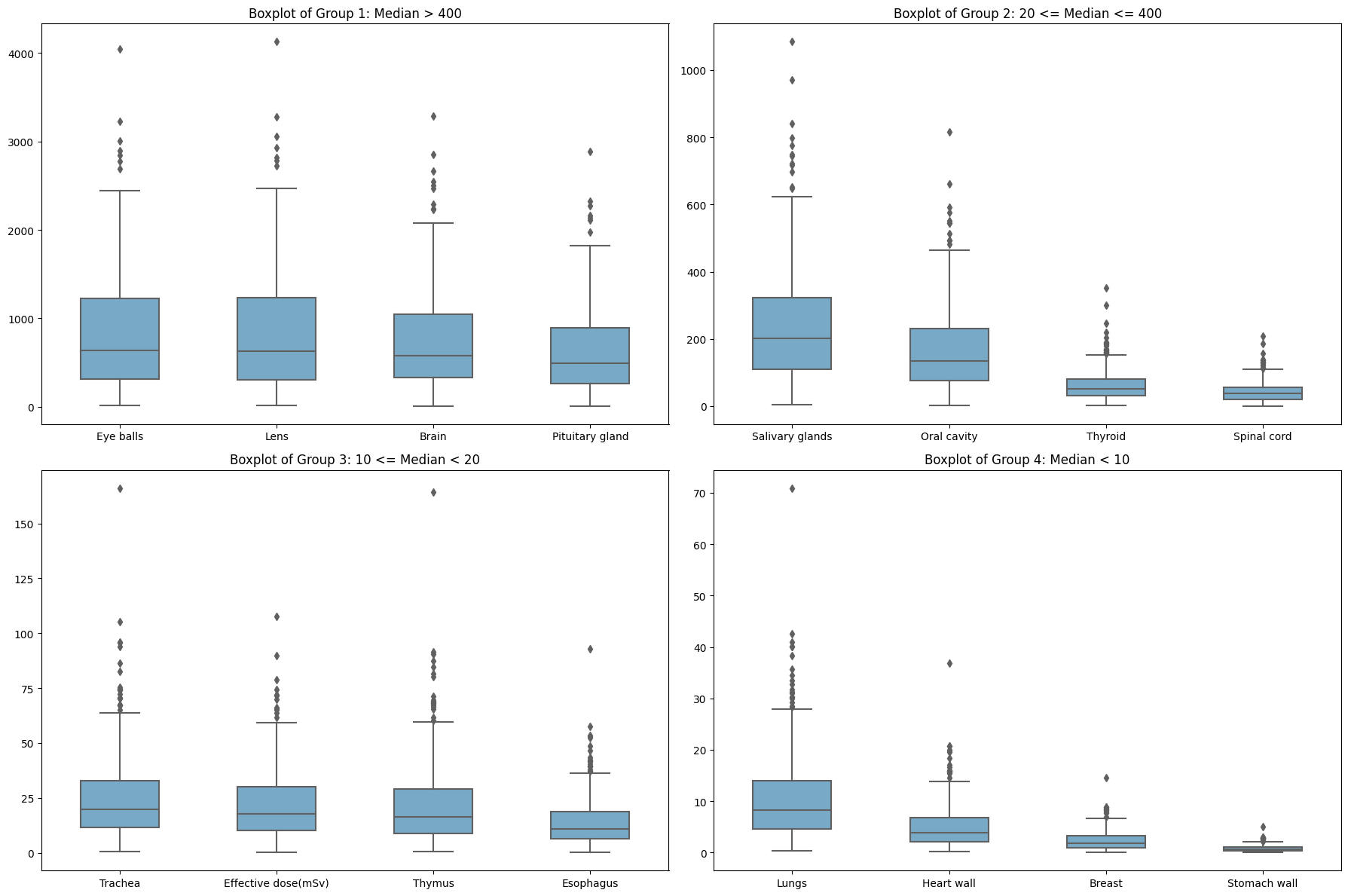


Median > 400 = body parts with median radiation exposure > 400 millisieverts

Y axis = median radiation exposure, x axis = body part exposed
