# Supplementary material for: Cumulative Radiation Exposure Post Aneurysmal Subarachnoid Haemorrhage
Source: Clin Neuroradiol. 2025 Mar 31;35(3):559–64. doi: 10.1007/s00062-025-01513-8 (PMC12454507; doi:10.1007/s00062-025-01513-8)
Supplement: Supplementary file 3 — Supplementary Table 1: Number of different types of investigations performed and the DLP for each scan type. COW = Circle of Willis, DLP = Dose length Product [file 62_2025_1513_MOESM3_ESM.docx]

Supplementary Table 1

| **Study Description** | **Count** | **Mean DLP per scan** | **Standard Deviation DLP** |
| --- | --- | --- | --- |
| **CT Brain** | 207 | 1166.97 | 936.84 |
| **CT Brain Perfusion – Code Stroke** | 36 | 4134.75 | 1052.36 |
| **CT Angiogram Head – COW** | 55 | 2113.40 | 1621.11 |
| **CT Perfusion - follow up** | 483 | 3742.53 | 595.00 |
| **CT Angiogram Carotid-COW with Plain Brain** | 7 | 1556.12 | 573.66 |
| **CT Angiogram Head and Carotid** | 1 | 1182.50 | N/A |

COW = Circle of Willis, DLP = Dose length Product
